# Veterinarians’ attitudes towards the treatment and surgical management of brachycephalic dogs – a survey among small animal veterinarians in Austria

**DOI:** 10.1186/s12917-026-05680-9

**Published:** 2026-07-01

**Authors:** Marlene Hebenstreit, Svenja Springer

**Affiliations:** Department of Interdisciplinary Life Sciences, Messerli Research Institute, University of Veterinary Medicine, Veterinaerplatz 1, Vienna, 1210 Austria

**Keywords:** Brachycephalic Obstructive Airway Syndrome, BOAS, Brachycephalic dogs, Small animal practice, Small animal veterinarians, Questionnaire study, Veterinary ethics

## Abstract

**Background:**

The rising popularity of flat-faced dog breeds has intensified discussions around brachycephaly and its associated health risks. The structural shortening of the skull coupled with a lack of a concurrent reduction in the volume of the soft tissues in these breeds often results in chronic health issues, notably Brachycephalic Obstructive Airway Syndrome (BOAS), which remains incurable but may be alleviated through corrective surgical interventions. In light of these challenges, veterinarians are increasingly called upon to provide informed guidance and support to pet owners navigating the complexities of brachycephalic breed care.

**Methods:**

Using an online questionnaire distributed to practising veterinarians in Austria (*N* = 395), we explored small animal veterinarians’ attitudes regarding the treatment and surgical management of brachycephalic dogs.

**Results:**

Veterinarians estimated that signs of BOAS are the primary presenting issue in 41–50% of the brachycephalic dogs they treat. According to the majority of surveyed veterinarians, snoring (96%) and difficulty breathing (92%) are frequently observed by owners. 40% of respondents disagreed that the treatment of brachycephalic dogs is interesting from a medical point of view. A further 30% stated that treating brachycephalic dogs is no different for them than treating other dogs. Surgical interventions for BOAS are recommended more frequently than they are performed, and veterinarians who perform surgery or work in surgical practices are significantly more likely to recommend these procedures (*p* < 0.001). Moreover, 70% of the veterinarians identified financial constraints as a common reason why owners decline surgical options. The severity of clinical signs of BOAS did not result in significant differences in the likelihood of recommending surgery (*p* = 0.363).

**Conclusions:**

Our study highlights the complexity surrounding attitudes to brachycephaly in dogs within the veterinary profession. While a significant proportion of veterinarians recognise the prevalence of BOAS signs, there is notable uncertainty regarding the treatment of these dogs. To address this, it is essential to enhance educational efforts aimed at both veterinarians and owners, ensuring a better understanding of the condition and its management.

**Supplementary Information:**

The online version contains supplementary material available at https://doi.org/10.1186/s12917-026-05680-9.

## Background

In recent years, the popularity of extreme brachycephalic dog breeds, such as Pugs, French Bulldogs, and English Bulldogs, has steadily increased [1, 2]. This trend, referred to by Packer et al. as the “Brachy Boom”, continues to gain momentum and draws significant attention to the health issues associated with brachycephaly in animal welfare discussions [3]. This growing concern is exemplified by campaigns in various countries, notably the British Veterinary Association’s #breedtobreathe initiative, which has been active since 2018 [4].

The intentional shortening of the skull in specific dog breeds, a consequence of decades of selective breeding [1], has led to a range of lifelong health issues known collectively as Brachycephalic Syndrome [1, 5, 6]. This syndrome encompasses a variety of conditions that disproportionately affect brachycephalic dogs compared to other breeds, stemming from anatomical deformities in critical areas such as the airways, gastrointestinal tract, teeth, eyes, middle ear, brain, and spine [2, 3, 7, 8]. Notably, the deformities in the upper airways represent not only a well-known, but also a prevalent and concerning issue in brachycephalic dogs [6], commonly referred to as Brachycephalic Obstructive Airway Syndrome (BOAS) [3]. The underlying problem stems from the disproportionate reduction of soft tissues relative to the shortening of the skull in affected animals. This imbalance results in a significant narrowing of the upper airways, characterised by features such as constricted nostrils, an elongated soft palate, and an enlarged tongue [2, 3, 9]. Consequently, various complications may arise, including laryngeal or bronchial collapse and tonsillar hypertrophy [2, 3]. The impaired respiratory function in dogs suffering from BOAS leads to dyspnoea, often presenting as snorting. This compromised breathing can result in a range of serious consequences, including reduced oxygen supply, decreased physical performance, heat intolerance, hyperthermia, cyanosis, collapse, syncope, and sleep disturbances [10]. A study by Riecks et al. retrospectively reviewed 62 cases of dogs that underwent surgery at Ohio State University Veterinary Teaching Hospital due to brachycephalic syndrome and found that snoring was the most common preoperative clinical sign (58.1%), followed by decreased physical performance (33.9%), and dyspnoea (29,0%) [11]. In contrast, syncope was observed least frequently at 12.9% [11].

In recent decades, medical approaches to alleviate BOAS signs have been developed against the backdrop of clinical problems. These can be either conservative or invasive, depending on the severity of clinical signs, through weight management, modification of activity and environmental conditions, as well as medication or surgical intervention [5, 11–13]. Surgical interventions aim to reduce airway resistance and can be performed on various problem areas, such as the pharynx, larynx, nostrils, or nasal cavity [14]. The surgical approach must be tailored to each individual patient, as there are numerous options for surgical correction. The prognosis of surgical correction varies depending on the patient, whereby the literature indicates a marking improvement of clinical signs associated with brachycephaly with a striking reduction in life-threatening events by 90% in dogs treated surgically [15]. However, despite this improvement, in another study respiratory function remains compromised in 60% of treated dogs [14]. Corrective surgery therefore cannot aim to cure BOAS but instead provide a better quality of life in most cases [13]. But although studies suggest that animals experience less suffering following surgical procedures [14, 15], there remains a lack of understanding regarding the threshold of severity of clinical signs at which veterinarians recommend surgical intervention for BOAS in clinical practice.

Veterinarians are not only trained to diagnose and manage health problems related to brachycephaly through medical and surgical treatments aimed at enhancing the quality of life for affected dogs. They are also expected to offer clear, evidence-based guidance in the public discourse surrounding brachycephaly. In Germany, for example, the Veterinary Chamber of Berlin, in cooperation with the State Office for Health and Social Affairs, has created posters entitled “Lifestyle or Living Beings” to raise awareness about brachycephaly [16]. Furthermore, in Austria, the government recently established a commission on harmful breeding practices, consisting of veterinary experts, to provide more clarity and enable more effective implementation of the national animal welfare law [17].

This responsibility to offer insight poses a challenge not only for the veterinary profession as a whole but also for individual practitioners. The need to provide informed guidance while promoting animal health requires a balanced approach. Veterinarians must have not only medical expertise but also the ability to communicate complex topics clearly, raising awareness about the issues surrounding brachycephaly and facilitating informed decision making [2]. As a result, veterinarians may find themselves navigating a landscape of conflicting interests when caring for brachycephalic patients. Their primary goal is to alleviate the suffering of animals experiencing signs of BOAS, a condition fundamentally influenced by human actions through targeted breeding in recent decades [1]. This can pose a stressful situation for practising veterinarians [18].

As a theoretical review by Fawcett et al. describes, veterinarians operate within a service-oriented framework where they are expected to respect the subjective perceptions of pet owners while also effectively communicating appropriate veterinary recommendations [2]. For example, when a veterinarian identifies the short snout of a brachycephalic dog as a contributing factor to health issues, they inadvertently critique a characteristic that many owners find particularly charming [2]. This situation may lead veterinarians to worry that raising such concerns could be perceived as judgemental, potentially alienating both brachycephalic dog owners and breeders [18, 19].

Hence, clinical decision-making in the management of brachycephalic dogs is influenced by a range of factors and should be considered within a broader context. Surgical interventions can provide symptomatic relief and improve quality of life in affected animals, but they do not address the underlying conformational traits associated with selective breeding. And even though several countries are aiming to control or improve the breeding of brachycephalic dogs, veterinarians must deal with the current number of patients suffering from BOAS. In this context, the availability of surgical expertise and services, along with the characteristics of the patient population, can significantly influence case presentations and treatment recommendations. For instance, pet owners seeking surgical management may preferentially consult clinics that offer these specialized procedures. Moreover, veterinarians with specific training and experience in BOAS surgery are more likely to consider and recommend such interventions, as they frequently witness positive outcomes and improvements in treated dogs compared to their colleagues.

Consequently, the treatment of brachycephalic dogs is a complex issue. This study focuses specifically on current clinical management practices and veterinarians’ attitudes regarding surgical interventions. Given the complexities faced by veterinarians in treating brachycephalic dogs, it is crucial that we more thoroughly incorporate their perspectives and experiences into research. Therefore, this study aims to investigate the frequency of BOAS signs as the primary reason for veterinary visits among brachycephalic dogs in Austria. It also explores how small animal veterinarians perceive the treatment of a brachycephalic dog and how often they recommend, perform, or refer for surgical correction and the severity of clinical signs that lead to these recommendations. Furthermore, the study examines the extent to which the costs associated with BOAS surgery influence pet owners’ decisions to refuse surgical intervention, according to the veterinarians’ assessments.

To achieve these objectives, we have formulated the following five research questions:

1. What percentage of brachycephalic dogs in veterinary care present with BOAS signs as the main reason for consultation, and how frequently do individual BOAS signs occur?
2. How do veterinarians perceive the treatment of brachycephalic dogs, and to what extent do aspects related to the veterinarian and the practice impact their perceptions?
3. How frequently do small animal veterinarians recommend surgical corrections to alleviate BOAS signs, how often are these procedures actually performed, and to what extent do factors related to the veterinary practice and the veterinarian influence the frequency of recommendations and performance of surgical corrections?
4. What are the most prevalent reasons that owners decide against surgery according to veterinarians?
5. What level of severity of clinical signs leads small animal veterinarians to recommend surgery to treat BOAS?

## Materials and methods

### Recruitment process

The present study was aimed at veterinarians practising in Austria. The Austrian Veterinary Chamber (ÖTK) supported the recruitment of study participants by sending invitations to participate in the study to its members via e-mail. The initial invitation to participate in the survey was sent to 4,885 ÖTK members on 16th September 2024. A second email was sent to 4,898 ÖTK members on 7th October 2024. The invitation and reminder emails contained general information, such as the aim of the study and the duration of the survey, as well as information about the approval of the study and the rights of study participants. The survey was closed on 11th November 2024. The study has been approved by the Ethics Committee of the Medical University of Vienna before the recruitment process began.

### Study participants

Completed questionnaires were returned by 395 Austrian veterinarians, giving a response rate of 8.1%. As the present study focuses on the treatment and surgical management of brachycephalic dogs (section  2.1 of the questionnaire), we only included respondents who indicated working at least 50% in small animal practice. This study therefore includes 317 small animal veterinarians residing in Austria. Detailed information about the socio-demographic and practice-specific factors for the study population is listed in Additional File 1.

### Survey development

Items related to the medical treatment of brachycephalic dogs were developed based on a literature review of the various issues associated with this disease [1–3, 5, 10, 20, 21]. The questionnaire was developed in German and underwent two stages of pre-testing to identify whether the content led to uncertainties or misunderstandings by the respondents. In the first step, cognitive interviews [22, 23] were conducted with four Austrian small animal veterinarians. In the second step, an online pre-test phase was conducted with nine Austrian veterinarians. All comments that could improve the quality of data were considered and incorporated into the final version of the questionnaire.

### Survey measurements

In total, the questionnaire consisted of five sections (Additional File 2) and was designed so that questions could be skipped, enabling respondents to progress even if they had not completed all of the questions in a previous section. Since only two sections providing insights into the clinical management of brachycephalic dogs are relevant to the present study, these are described in detail in the following paragraphs. The associated questions from the mentioned sections are also presented in Table 1.

**Table 1:** Relevant questions from the questionnaire in section 2

Section of the questionnaire	Number of the question in the study	Question
Section 2	15.	What percentage of the dogs that are presented to you as patients in your work are brachycephalic?
Section 2	17.	Attitude statements towards the treatment of brachycephalic dogs
Section 2.1	19.	In what percentage of the brachycephalic dogs that you care for as patients are symptoms of BOAS the main reason for presentation?
Section 2.1	20.	How often do the following symptoms of BOAS occur in the brachycephalic dogs you care for as patients?
Section 2.1	21.	What percentage of brachycephalic dogs with symptoms of BOAS that you care for as patients do you recommend surgery to relieve symptoms?
Section 2.1	22.	What percentage of brachycephalic dogs with symptoms of BOAS for whom you recommend surgery actually undergo surgery?
Section 2.1	23.	Where are surgeries performed to relieve symptoms of BOAS of your patients?
Section 2.1	24.	If pet owners reject a surgery you recommend to alleviate BOAS symptoms, to what extent do the following factors influence the rejection on the part of the pet owners?

The first section contained 13 closed-ended questions on socio-demographic and practice-specific factors. The second section included items on the medical care of brachycephalic dogs, with section 2.1 focusing on issues related to BOAS. One question in section 2 asked about the percentage of brachycephalic dogs presented to veterinarians. Answer options ranged from “0%” to “1–10%”, “11–20%”, and so on up to “91–99%” and “100%”. The answer option “I don’t know” was also available. Furthermore, four statements were presented to explore veterinarians’ attitudes towards the treatment of brachycephalic dogs by focusing on positive (e.g. a medical interest in the treatment) and negative effects (e.g. leading to a lack of understanding or triggering anger about owning a brachycephalic dog). Respondents could indicate their level of agreement with each statement using a 7-point Likert scale [24]: 1 “Strongly disagree”, 2 “Disagree”, 3 “Somewhat disagree”, 4 “Neutral (neither agree nor disagree), 5 “Somewhat agree”, 6 “Agree”, and 7 “Strongly agree”. In addition, “I don’t know” was also given as an option.

A short introductory text at the beginning of section 2.1 explained the definition of BOAS, typical signs, and methods for BOAS screening. This was followed by a question about the percentage of brachycephalic dog patients that have BOAS signs as their main reason for presentation. Again, answer options ranged from “0%” to “1–10%”, “11–20%”, and so on up to “91–99%”, and “100%”. The answer “I don’t know” was also available as an option. Participants were then presented with ten signs of BOAS that can occur in brachycephalic dogs, e.g. difficulty breathing, heat intolerance, snoring, or regular collapse. By providing the answer options “Not at all”, “Rarely”, “Occasionally”, ‘Frequently’, “Very frequently”, and “I don’t know”, veterinarians were able to indicate the frequency of signs observed in their treated patients. In addition, this section included a question about the percentage of patients with BOAS signs where veterinarians recommend surgery to relieve the clinical signs and the percentage of patients that actually undergo the recommended procedure. Answer options ranged from “0%” to “1–10%”, “11–20%”, and so on up to “91–99%”, and “100%”. The answer “I don’t know” was also available as an option.

A further question asked where surgery to relieve signs of BOAS was performed. In total, three answer options were provided: (1) “I perform the surgery myself in the practice/clinic where I work”, (2) “One of my colleagues performs the surgery in the practice/clinic where I work”, and (3) “I refer the patient to another practice/clinic where the surgery is performed”. The answer option “Other” was also provided. To gain insights of veterinarians’ perceptions of why pet owners refuse surgery, we presented eight possible reasons varying from limited financial resources to fear of complication and negative experiences with other animals that have undergone this therapy to the veterinarians and asked to what extent they influence pet owner’s refusal of surgery. Answer options were “Not at all”, “Very little”, “Moderately”, “Quite strongly”, “Very strongly”, and “I don’t know”.

Lastly, section 2.1 included a case vignette [25] in which the study population was randomly allocated to receive different information (henceforth referred to as scenarios) about the patient’s clinical signs. In all three scenarios, the participants were given a description of a patient with varying signs of BOAS. The first scenario was presented to 35.5% (*n* = 111) of all the small animal veterinarians:

*A dog owner comes to you with her two-year-old pug Maxi. Maxi’s difficulty breathing is immediately noticeable. In addition*,* the dog owner reports that she can hardly go out of the house with Maxi on warm days and that hesnores terribly loudly while sleeping*. *Based on the symptoms and further diagnostics*,* you will diagnose BOAS in Maxi. How would you proceed?*

In the second scenario, the difficulty breathing remains, but the second sign was changed to “his tongue even turns blue during exertion” (= cyanosis). This scenario was presented to 29.97% (*n* = 95) of the veterinarians. The third scenario was presented to 34.38% (*n* = 109) of veterinarians and included the sign “while walking, he had already collapsed several times exhausted and even lost consciousness the last time”. In each scenario, the respondents could choose between the following options: “I would recommend surgery to relieve the symptoms of BOAS”, “I would not recommend any surgical intervention to relieve the symptoms of BOAS”, and “Other”.

### Data analysis

The online survey was set up using the survey software Alchemer, and IBM SPSS Statistics version 30.0 (IBM^®^ SPSS^®^ Statistics, Chicago, IL, United States) was used for all analyses. Univariate descriptive statistics were presented in tables, figures, or text. For bivariate analysis, Chi-Square Tests were performed to test whether the frequency distribution between the veterinarians recommending surgery differed among the three case scenarios. We considered p-values below 0.05 as statistically significant.

To examine the effect of veterinarian-related factors on different perceptions of treating brachycephalic dogs, we performed four ordinal regression analyses. The dependent variables were: “The treatment of a brachycephalic dog…” (1) “…is interesting from a medical perspective”, (2) “…leads to a lack of understanding about owning such a dog”, (3) “…triggers anger in me about owning such a dog”, and (4) “…is no different from treating other dogs”. An ordinal scale was used for the dependent variables (1 = Agreement, 2 = Neutral, and 3 = Disagreement). The six predictor variables included in the regression analyses were: (i) gender (1 = male; 2 = female), (ii) veterinarians’ work experience (range: 1–51 years), (iii) level of urbanisation (1 = urban region; 2 = rural region), (iv) owning a dog (1 = yes, 2 = no) and (v) percentage of brachycephalic dogs among patients treated in practice (1 = 0, 2 = 1–10%, up to 12 = 100%). The final variable included in the regression analyses indicated whether veterinarians perform the surgery themselves, whether it is conducted in the clinic where they work, or whether they need to refer the patient elsewhere (1 = operation performed by themselves or in the clinic by colleagues; 2 = refer to a clinic/other).

Two ordinal regression analyses were performed to examine the effect of veterinarian-related factors on (1) how frequently surgical intervention was recommended and (2) how frequently surgical intervention was actually performed based on veterinarians’ recommendations to alleviate BOAS signs. The dependent variables were represented on an ordinal scale (1 = 0% of cases, 2 = 1–10% of cases, up to 91–100% of cases), where veterinarians’ estimated how often they recommend surgical intervention to alleviate BOAS signs. The six predictor variables included in the regression analyses were: (i) gender (1 = male; 2 = female), (ii) veterinarians’ work experience (range: 1–51 years), (iii) level of urbanisation (1 = urban region; 2 = rural region), and (iv) level of agreement with the statement “The treatment of a brachycephalic dog is interesting from a medical point of view” and (v) “The treatment of a brachycephalic dog is no different from treating other dogs”. As a relatively high percentage of veterinarians chose the answer option “I don’t know” regarding these statements (32.8% and 11.5%), we decided to include these respondents in the regression analyses as follows: 1 = disagreement, 2 = agreement, 3 = neutral/I don’t know. The final variable included in the regression analyses indicated whether veterinarians perform the surgery themselves, whether it is conducted in the clinic where they work, or whether they need to refer the patient (1 = operation conducted by themselves or in the clinic by colleagues; 2 = refer to a clinic/other).

## Results

### Estimated percentage of treated brachycephalic dogs and treated brachycephalic dogs with BOAS signs as the main reason for consultation

Table 2 presents the veterinarians’ estimates of the relative numbers of brachycephalic dogs among those they treat and brachycephalic dogs with BOAS signs as the main reason for consultation. The majority (70%) of veterinarians indicated that between 1% and 20% of the dogs they treat are brachycephalic. In addition, 32.2% of veterinarians stated that BOAS signs were the main reason for consultation in over 60% of their brachycephalic dog patients.

**Table 2:** Estimated number of treated brachycephalic dogs (*N* = 279) presenting due to BOAS signs (*N* = 261)

Treated brachycephalic dogs	Estimated percentage of patients	Treated brachycephalic dogs with BOAS signs as the main reason for consultation
1 (0.4)	None	9 (3.4)
196 (70.3)	1–20%	68 (26.1)
67 (24.0)	21–40%	49 (18.8)
5 (1.8)	41–60%	43 (16.5)
1 (0.4)	> 60%	84 (32.2)
9 (3.2)	I don’t know	8 (3.1)

Number (percentage)

### Estimated occurrence of BOAS signs in treated brachycephalic dogs

**Fig. 1:**
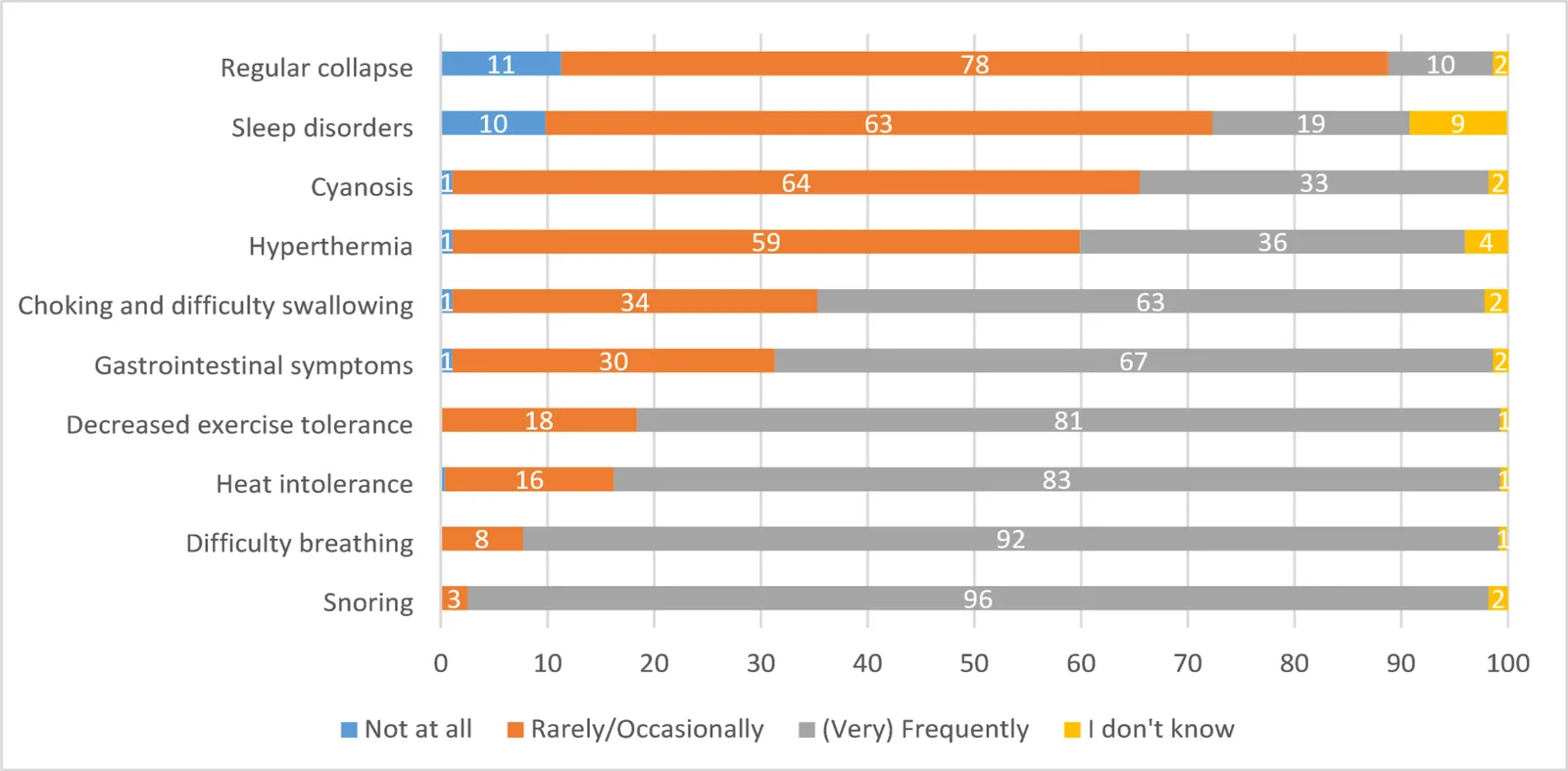
Estimated occurrence of BOAS signs in treated brachycephalic dogs according to veterinarians (*N* = 271–275) (in percentage)

Almost all (95.7% and 91.5%, respectively) of the surveyed veterinarians frequently identified snoring and difficulty breathing in their patients (Fig. 1). In contrast, only 10% of veterinarians reported the clinical sign of collapse.

### Veterinarians’ perception of the treatment of brachycephalic dogs

Even though almost half of the respondents disagreed that treating a brachycephalic dog is interesting from a medical point of view (40.4%), for nearly one third of veterinarians the perception does not differ significantly from the treatment of other dogs (30,0%) (Table 3). A considerable percentage of respondents chose the option “I don’t know”, with nearly one-third of the veterinarians (32.8%) uncertain about whether they find treating brachycephalic dogs interesting. However, 47.3% of owners agreed that this uncertainty contributes to a lack of understanding about owning such a dog.

**Table 3:** Veterinarians’ perception of treating brachycephalic dogs

The treatment of a brachycephalic dog…	Level of agreement	*N* = 313–317
▪…is interesting for me from a medical perspective.	Disagreement	127 (40.4)
	Neutral	29 (9.2)
	Agreement	55 (17.5)
	I don’t know	103 (32.8)
	Mean ± Std.*	3.34 ± 2.07
▪ …leads to a lack of understanding about owning such a dog.	Disagreement	76 (24.0)
	Neutral	71 (22.4)
	Agreement	150 (47.3)
	I don’t know	20 (6.3)
	Mean ± Std.*	4.25 ± 1.11
▪ …triggers anger in me about owning such a dog.	Disagreement	141 (44.8)
	Neutral	38 (12.1)
	Agreement	73 (23.2)
	I don’t know	63 (20.0)
	Mean ± Std.*	3.63 ± 1.73
▪ …is no different for me than treating other dogs.	Disagreement	148 (47.3)
	Neutral	35 (11.2)
	Agreement	94 (30.0)
	I don’t know	36 (11.5)
	Mean ± Std.*	3.77 ± 2.13

Number (percentage) *1 = strongly disagree; 2 = disagree; 3 = somewhat disagree; 4 = neutral (neither agree nor disagree); 5 = somewhat agree; 6 = agree; 7 = strongly agree. The response option ‘I don’t know’ was excluded when calculating the mean.

### What impacts veterinarians’ perceptions of treating brachycephalic dogs?

Veterinarians with a decrease in years of work experience were more likely to agree that the treatment of a brachycephalic dog leads to a lack of understanding about owning such a dog compared to veterinarians with an increase in years of work experience (*p* = 0.002) (Additional File 3).

### Veterinarians’ recommendations for and actual performance of surgical interventions to alleviate BOAS signs

Table 4 presents how often small animal veterinarians recommend surgery to alleviate the clinical signs of BOAS and how often surgery is subsequently performed. The results indicate that 44.0% of veterinarians reported recommending surgical intervention for more than 60% of dogs presenting with BOAS. However, when considering the actual performance of surgical interventions based on these recommendations, 46.1% of respondents reported that only 1% to 30% of their recommendations result in surgery.

**Table 4:** Veterinarians’ recommendations for (*N* = 271) and actual performance of surgery (*N* = 256) to alleviate BOAS signs

Recommendation for surgical intervention to alleviate BOAS signs	Estimated percentage of patients	Actual performance of surgical intervention to alleviate BOAS signs
7 (2.6)	None	11 (4.3)
80 (29.5)	1–30%	118 (46.1)
47 (17.3)	31–60%	43 (16.8)
69 (25.5)	61–90%	35 (13.7)
50 (18.5)	91–100%	11 (4.3)
18 (6.6)	I don’t know	38 (14.8)

Number (percentage)

### What impacts the frequency of recommendations for and actual performance of surgical intervention to alleviate BOAS signs?

Veterinarians who perform the surgery themselves or work in a practice/clinic where a colleague does the surgery tended to recommend the procedure more often and perform a higher rate of surgical intervention compared to veterinarians who need to refer the patient to another clinic (*p* < 0.001) (Additional File 4).

Furthermore, female veterinarians and those with more years of work experience performed a significantly lower rate of recommended surgical intervention compared to their male colleagues (*p* = 0.004) and those with fewer years in practice (*p* = 0.003) (Additional File 4).

### Veterinarians’ perceptions of owners’ reasons for refusing surgical intervention

**Fig. 2:**
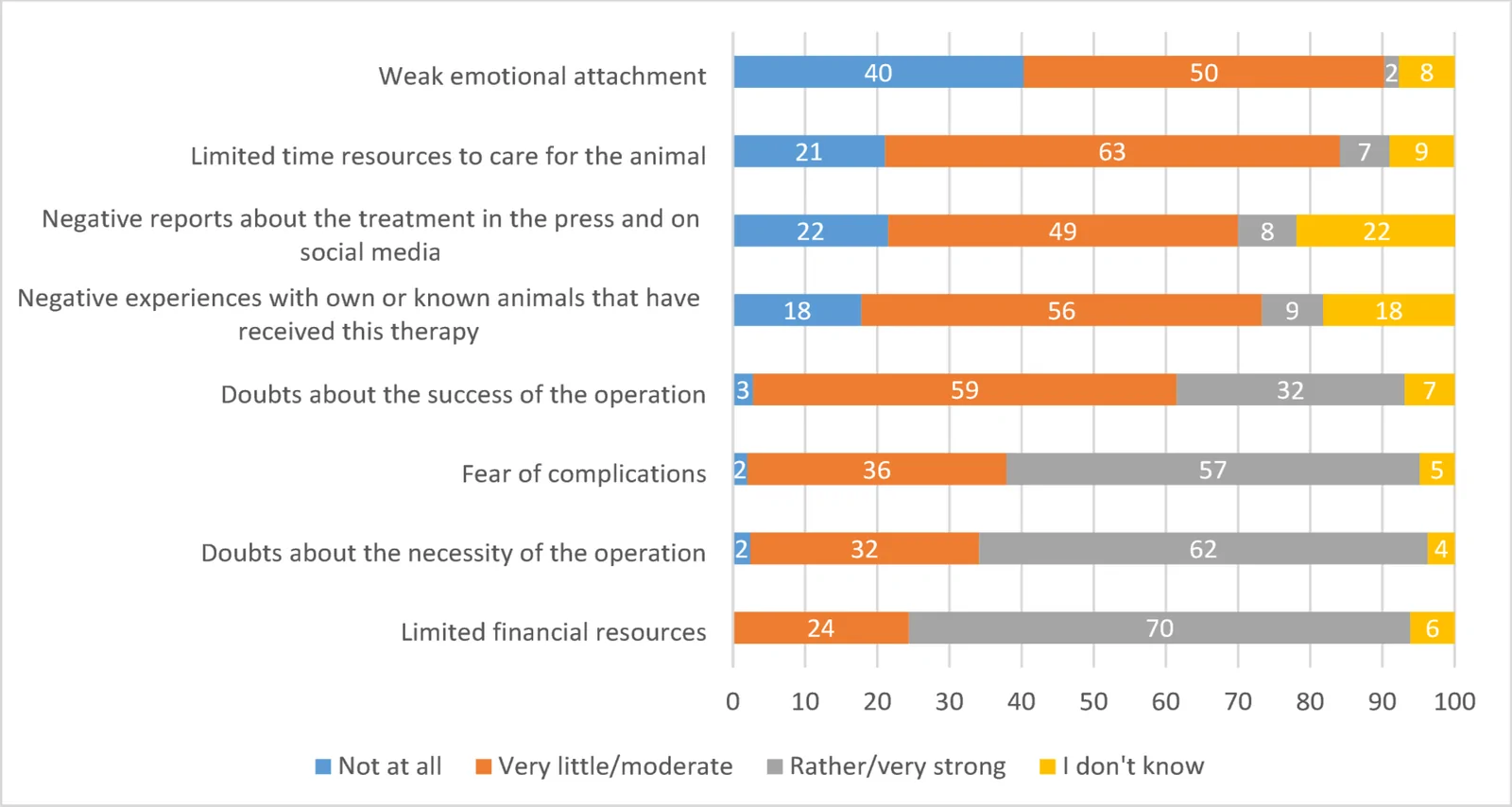
Reasons that influence owners’ decisions against recommended BOAS surgery according to veterinarians (*N* = 246–248) (in percentage)

Figure 2 presents results regarding veterinarians’ perceptions of the factors influencing dog owners’ decisions to refuse recommended surgical interventions. The most significant reasons identified were financial limitations and doubts about the necessity of the operation, with 69.5% and 62.2% of veterinarians citing these reasons, respectively. In contrast, emotional attachment and limited time resources play a minor role according to veterinarians, with only 2.0% and 6.9% of respondents indicating these as strong reasons for refusal.

### Impact of clinical signs to recommend surgical intervention

**Fig. 3:**
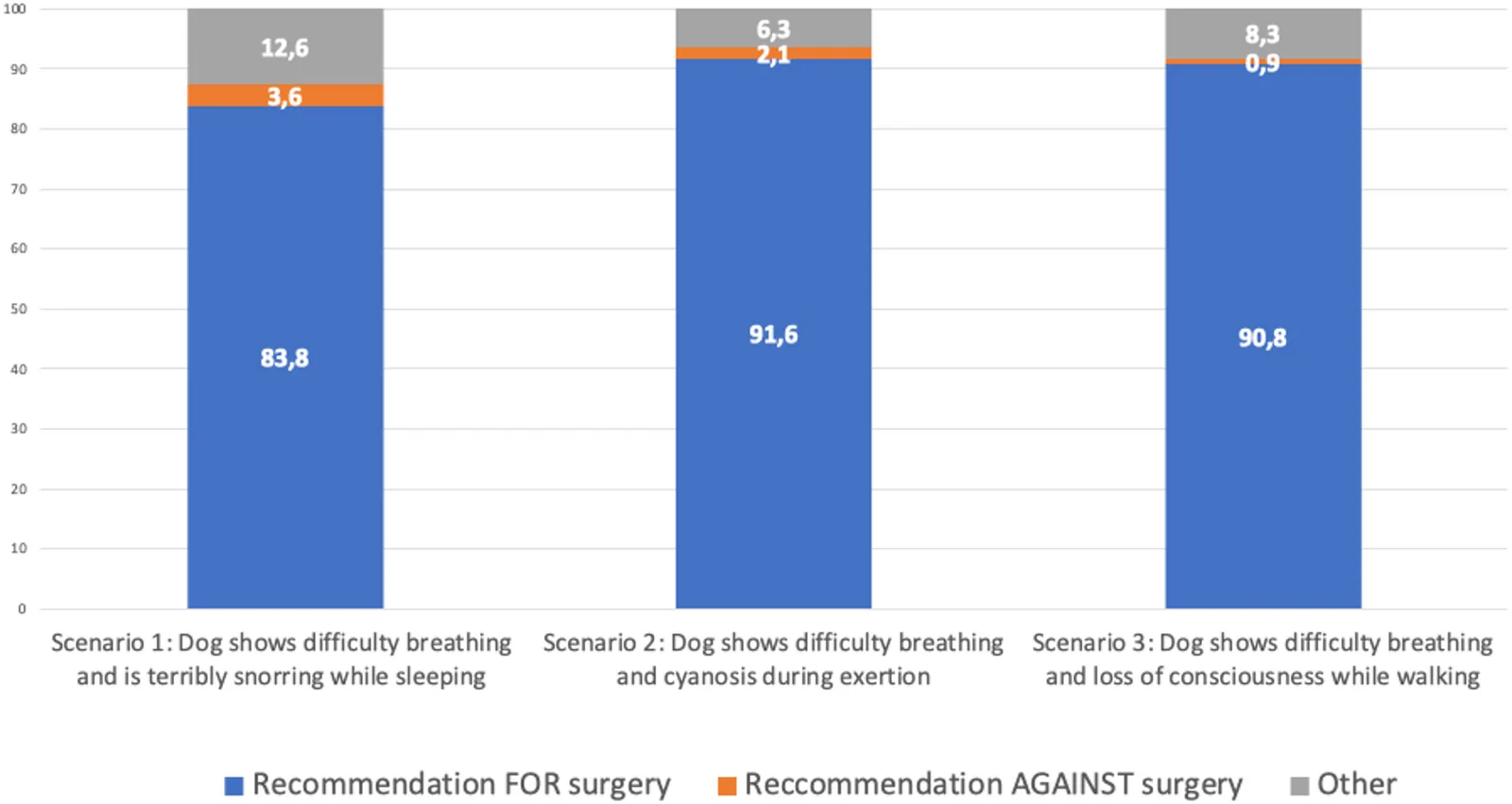
Distribution of veterinarians’ recommendations for performing surgical intervention to alleviate BOAS signs (*N* = 315; scenario 1: *n* = 111 (35.2%), scenario 2: *n* = 95 (30.2%), scenario 3: *n* = 109 (34.6%)) (in percentage)

As shown in Fig. 3, variation in the severity of BOAS signs in the scenarios had little influence on the small animal veterinarians’ responses. In all three cases, the majority of veterinarians decided to recommend surgery. Even though fewer veterinarians (scenario 1: 83.8%) recommended surgery for mild signs compared to moderate and severe signs (case B: 91.6%, case C: 90.8%), the comparative analysis showed no significant difference (Chi [2] = 2.034, *p* = 0.363).

## Discussion

The study provides insights into Austrian small animal veterinarians’ attitudes towards the treatment and surgical management of brachycephalic dogs. The results show that owning a brachycephalic dog provoked a lack of understanding in around half of the participating veterinarians, and those with less work experience felt this more than veterinarians with more years of professional experience. One possible explanation might be that younger veterinarians, who are likely to have encountered ethical discussions regarding brachycephaly during their education, seem to adopt a more critical stance towards the breeding and ownership of brachycephalic dogs compared to their more seasoned counterparts, who may not have had formal education into this topic. This observation implies that early exposure to these ethical considerations could lead to a deeper reflection on the implications of such practices. Additionally, it appears that owning a flat-faced breed can evoke a notable sense of discontent in a significant proportion of individuals. When asked whether treating a brachycephalic patient was interesting from a medical perspective, a third of veterinarians responded with “I don’t know”. This ambiguity may be attributable to the term “interesting,” which could have been perceived as insufficiently precise. Alternatively, the responses may reflect the uncertainty veterinarians feel regarding the treatment of flat-faced patients and how to deal with the issues surrounding brachycephaly in dogs. The number of flat-faced dogs is increasing steadily [1, 7], as are the number of research findings highlighting the link between brachycephaly and health consequences [2]. On the one hand, this opens up a new field and potential source of income in the area of small animal medicine [18]. On the other hand, however, it also raises questions about the owners’ level of knowledge regarding brachycephaly and the recognition of health problems in their brachycephalic animals [10, 26], which may cause frustration among veterinarians. Accordingly, a study that surveyed Danish owners of French Bulldogs indicated that they are less interested in information about the health of the breed before purchasing a dog than owners of other dog breeds [27].

The results reveal a lack of recognition of clinical signs linked with brachycephaly by dog owners. On the one hand, according to veterinarians, six out of ten listed signs of BOAS occur frequently to very frequently in brachycephalic dogs. But on the other hand, more than a quarter of the veterinarians stated that signs of BOAS are the main reason for presentation in only 1–20% of their flat-faced patients. This indicates that although BOAS signs occur frequently, pet owners may not consider them a reason to take their pet to a veterinarian. In this context, the literature refers to a lack of knowledge and awareness among pet owners about possible signs associated with brachycephaly [10, 27]. A study conducted in the UK, for example, shows that 58% of owners of brachycephalic dogs do not recognise the signs of brachycephalic syndrome and instead consider them to be “normal” for the breed [10]. The major problem here is that owners of brachycephalic dogs do not consult a veterinarian if they fail to recognise health problems, meaning that the animal cannot receive adequate medical treatment. As a result, this promotes the development of chronic diseases [10].

In a study among dog owners in English-speaking countries, 20% of respondents stated that their dog had undergone surgery due to its morphology, with nasal dilation being the most common procedure [20]. The results of the present study show that surgical intervention to alleviate the clinical signs of BOAS is recommended by veterinarians much more frequently than surgery is actually performed. Furthermore, we identified that veterinarians who perform such operations themselves or work in a practice or clinic where they are performed are significantly more likely to recommend surgery than veterinarians who refer patients elsewhere. On the one hand, this significant difference could possibly be explained by the presence of medical expertise in the practice or clinic, because of which owners seeking surgical management may preferentially consult them. On the other hand, also the financial gain that results from it could be a possible explanation. In this context, Fawcett et al. describe a possible conflict of interest among veterinarians who derive their income from treating BOAS and the primacy of financial interests over medical considerations [2].

Another finding of the study suggests that veterinarians with more years of professional experience perform BOAS surgery less frequently. As the topic of extreme breeding and related health problems is currently at its peak and became of particular interest in the last 10 years, this could indicate that less experienced – and hence younger – veterinarians may be more aware of BOAS and its possible consequences since the topic was likely to have been raised during their studies. Consequently, they may be better equipped to deal with the challenges of confronting owners of brachycephalic dogs about the issue of BOAS – including on an ethical and communicational level – as education in these areas is becoming increasingly important at veterinary universities [28]. Unlike the younger generation, who have grown up with an awareness and a certain condemnation of breeding flat-faced breeds, more experienced veterinarians were not socialised into the ethical discussion surrounding brachycephaly in this way. Thus, older veterinarians have certainly been aware of the problems related to brachycephaly since the publicity began, but are perhaps not used to the topic being discussed as critically as it is today. In conclusion, it is fair to say that a recommendation of surgery to alleviate the clinical signs of BOAS not only depends on the patient, but also on the veterinarian consulted and of course, the animal owner.

The decision about whether to perform BOAS surgery after a recommendation by a veterinarian ultimately lies with the animal’s owners. According to the veterinarians’ perception, the main reason pet owners reject surgical correction is due to limited financial resources. This once again highlights the importance of financial aspects regarding the surgical treatment of BOAS, not only in relation to veterinarians, but also the animal owners. The cost of surgery as a reason for refusal is probably also related to a lack of knowledge about brachycephalic dogs among owners, who underestimate not only the potential health problems caused by brachycephaly when purchasing a brachycephalic dog, but also the increased financial costs [2]. For example, a study by Packer et al. found that more than one-fifth of brachycephalic dog owners surveyed had underestimated the veterinary costs involved [20]. Neglecting this aspect can lead to brachycephalic dogs not receiving adequate treatment due to a lack of financial resources or an unwillingness to pay for medical procedures.

Veterinarians also indicated that the second most common reason given by owners for refusing surgery to alleviate the clinical signs of BOAS was that they doubted the necessity of the operation. This could again point to a lack of awareness and recognition of the signs of brachycephalic syndrome by owners [10]. This lack of objectivity among owners of brachycephalic dogs is also evident in a Danish study in which 64% of French Bulldog owners surveyed stated that although French Bulldogs are generally less healthy than other dogs, their own dog is healthier than others of the same breed. As a consequence, if the clinical signs are not recognised or are not considered worthy of action, surgery is also unlikely to be considered useful [29]. In this context, however, it should be noted that the literature indicates a marking improvement of clinical signs associated with brachycephaly with a striking reduction in life-threatening events by 90% in dogs treated surgically [15], and BOAS surgery can therefore provide a better quality of life in most cases [13].

Fear of complications was the third most common reason to refuse BOAS surgery. In this regard, the 1.57 times increased intra-anaesthetic risk of complications in brachycephalic dogs compared to mesocephalic dogs, as well as the 4.33 times increased post-anaesthetic risk of complications must be mentioned [30]. For instance, in a retrospective study by Filipas et al., 199 clinical records were analysed and hypoxaemia was observed postoperatively in 5% of cases, dyspnoea requiring tracheal re-intubation in 7%, dyspnoea requiring tracheostomy in 5%, and aspiration pneumonia in 6% of cases [31]. Nevertheless, considering the potential improvement in the quality of life of affected dogs after surgery, it must be questioned whether these numbers justify refusing surgery and the resulting lifelong suffering.

We hypothesised that veterinarians would be more likely to recommend surgery to alleviate the clinical signs of BOAS in cases of more severe signs than in cases of mild signs. However, comparative analyses of the results of our case vignettes showed that the different clinical signs mentioned had little influence on the veterinarians’ responses – the majority (90%) of participants recommended surgery in all three cases. This refuted the assumption that the veterinarians’ responses would differ depending on the severity of the clinical signs described. Since a study by Filipas et al. specifies that severe BOAS increases the likelihood of postoperative complications [31], it is positive that the participating veterinarians would recommend surgery even in cases of relatively mild signs.

Although this study provides insights into BOAS-related perspectives of small animal veterinarians practising in Austria, certain limitations must be taken into account. While all veterinarians practising in Austria were contacted via the Austrian Veterinary Chamber, the response rate was only 8.1% (*N* = 395). For this paper, only answers from 317 small animal veterinarians were taken into consideration. Since there were many follow-up questions in the questionnaire (Additional File 2), the number of responses gradually decreased, which is why the response rates are well below 317.

Furthermore, a possible bias must be taken into account, as it is likely that only those veterinarians who are interested in the topic of brachycephaly in dogs participated in the questionnaire study. Although understanding of the questions was tested through cognitive interviews and written feedback, it is important to consider that the questions and answer options may not have been understood or interpreted in the same way by all participants. In addition, the assessment of BOAS classification in our study may underestimate the severity of the condition. Maxi’s clinical signs—difficulty breathing at rest, exercise intolerance, and heat intolerance—may suggest a higher BOAS grade than previously categorized [21, 32]. This overlap in BOAS grading among cases in Group A indicates that we may not have fully captured the complexity of these conditions. Future research should focus on this issue to better reflect the severity of symptoms observed in brachycephalic dogs.

Further research is warranted to advance our understanding of brachycephaly in dogs. As this study was limited to veterinarians in Austria and tailored to the structure of this country, it would be of interest to investigate veterinarians’ perceptions of the treatment of brachycephalic dogs in other countries as well, in order to allow for an international comparison. Furthermore, it is essential to explore the perspectives of other key stakeholders, such as breeders and owners of brachycephalic dogs, in greater detail. Additionally, it is crucial to identify and implement optimal strategies for modifying breeding practices to ensure healthier offspring in brachycephalic breeds. Addressing these areas will contribute significantly to the overall welfare of these animals and promote more responsible breeding practices.

## Conclusion

Based on our findings we conclude that BOAS is a very common disease in brachycephalic dogs, yet it is comparatively rarely given as the main reason for presentation at the veterinarian. The most frequently reported BOAS sign by veterinarians are snoring and difficulty breathing. Regarding veterinarians’ perception of the treatment of brachycephalic dogs, nearly one third were uncertain about whether they found treating brachycephalic dogs interesting from a medical perspective, and half of veterinarians lacked an understanding about owning such a dog. Furthermore, we found that surgical treatment of BOAS is recommended much more often than it is actually performed, whereby veterinarians who perform surgery themselves or work in a practice/clinic where a colleague does the surgical intervention tend to recommend the procedure more often and perform surgery at a higher rate compared to veterinarians who need to refer the patient to another clinic. Refusal of surgery by the owners of brachycephalic dogs results most frequently from financial limitations and doubts about the necessity of the operation. Finally, we identified that the severity of BOAS signs had no significant influence on the recommendation of surgical treatment by veterinarians.

## Supplementary Information

Additional File 1: Data analysis of socio-demographic and practice-specific factors. Summary of the socio-demographic and practice-specific data of the study population, collected in the first section of the questionnaire study.

Additional File 2: Questionnaire. Complete questionnaire consisting of five sections, which was used to survey Austrian veterinarians on the topic of brachycephaly in dogs.

Additional File 3: Ordinal logistic regression analysis to examine the effect of socio-demographic and practice-related aspects on the veterinarians’ perception of treating brachycephalic dogs.

Additional File 4: Ordinal logistic regression analysis to examine the effect of veterinarian-related factors on the frequency of recommending surgical intervention to alleviate BOAS signs and ordinal logistic regression analysis to examine the effect of veterinarian-related factors on the frequency of actual surgical intervention based on the veterinarians’ recommendation to alleviate BOAS signs.

## Data Availability

The dataset supporting the conclusions of this article is included within the article and its additional files.
