# Supplementary material for: Veterinarians’ attitudes towards the treatment and surgical management of brachycephalic dogs – a survey among small animal veterinarians in Austria
Source: BMC Vet Res. 2026 Jul 1;22:594. doi: 10.1186/s12917-026-05680-9 (PMC13656368; doi:10.1186/s12917-026-05680-9)
Supplement: Supplementary file 1 [file 12917_2026_5680_MOESM1_ESM.docx]

**Data analysis of socio-demographic and practice-specific factors**

Marked in red: Questions addressed to all participants

Marked in green: Questions that were addressed exclusively to small animal veterinarians

1. **Age**

- n = 350
- missing = 45
- min = 24
- max = 83
- mean = 46.65
- median = 47.5
- Std. variation = 12,148
- IQR = 36; 56

Age Women

- n = 289
- missing = 31
- min = 24
- max = 76
- mean = 44.84
- median = 45
- Std. variation = 11,086
- IQR = 35; 54

Age Men

- n = 58
- missing = 13
- min = 29
- max = 83
- mean = 55.1
- median = 57
- Std. variation = 12,762
- IQR = 50.25; 60,25

2. **Sex**

- n = 394
- missing = 1
- male = 71 = 18%
- female = 320 = 81.2%
- diverse = 1 = 0.3
- I don't want to specify = 2 = 0.5%

3. **Status**

- n = 395
- missing = 0
- 177 = 44.8% Self-employed, full-time
- 35 = 8.9% Self-employed, part-time
- 98 = 24.8% Employee, full-time
- 78 = 19.7% employed, part-time
- 22 = 5.6% In Pension

4. **Years of professional experience**

- n = 391
- missing = 4
- min = 1
- max = 51
- mean = 17.89
- median = 18
- Std. variation = 11,471

5. **Institution**

- n = 395
- missing = 0
- 299 = 75.7% Veterinary practice
- 66 = 16.7% Veterinary Clinic
- 13 = 3.3% University Hospital
- 13 = 3.3% Mixed practice/clinic
- 1 = 0.3% Animal shelter in employment
- 5 = 1.3% Charity
- 40 = 10.1% Other

6. **Space (degree of urbanization)**

- n = 393
- missing = 2
- 113 = 28.8 % Large urban centre ( > = 100,000 inhabitants)
- 56 = 14.2% Urban centre (> = 30,000 and < 100,000 inhabitants)
- 60 = 15.3% Urban small centre (< 30,000 inhabitants)
- 120 = 30.5% Rural area central (accessibility to urban centre < 30 minutes)
- 40 = 10.2% Rural peripheral areas (accessibility to urban centre > 30 minutes)
- 4 = 1.0% I don't want to specify

7. **Number of veterinarians in practice/clinic**

- n = 386
- missing = 9
- min = 1
- max = 21
- mean = 4.92
- median = 3
- Std. variation = 5,625
- IQR = 1; 6

8. **Workspace**

- n = 395
- missing = 0
- 351 = 88.9 % Small animal medicine
- 53 = 13.4 % Livestock medicine
- 65 = 16.5 % Equine medicine
- 3 = 0.8% Laboratory Veterinary Medicine
- 4 = 1.0% Zoo Veterinary Medicine
- 23 = 5.8% Exotic medicine
- 23 = 5.8% Avian medicine
- 25 = 6.3% Authority
- 32 = 8.1% Food hygiene and food safety
- 2 = 0.5% Pharmaceutical Industry
- 18 = 4.6% Other

9. **Activity in the field of small animal medicine**

- n = 351
- missing = 0

| Specification | Answer |
| --- | --- |
| 10% | 12 (3,4%) |
| 20% | 6 (1,7%) |
| 30% | 9 (2,6%) |
| 40% | 7 (2,0%) |
| 50% | 25 (7,1%) |
| 60% | 5 (1,4%) |
| 70% | 10 (2,8%) |
| 80% | 15 (4,3%) |
| 90% | 37 (10,5%) |
| 100% | 225 (64,1%) |

**10. Additional qualification**

- n = 317
- missing = 0
- 37 = 11.7 % Specialist veterinarian
- 12 = 3.8% Diplomate (e.g. European or American College)
- 97 = 30.6% Doctor of Veterinary Medicine
- 1 = 0.3% PhD
- 187 = 59.0% I don't have any of the above qualifications.

**11. Department**

- n = 47
- missing = 2
- Anesthesia and pain management = 3 = 6.4%
- Ophthalmology = 4 = 8.5%
- Diagnostic imaging = 6 = 12.8%
- Surgery (orthopedics) = 11 = 23.4%
- Surgery (soft tissues) = 17 = 36.2%
- Dermatology = 3 = 6.4%
- Internal medicine = 12 = 25.5%
- Cardiology = 6 = 12.8%
- Neurology = 3 = 6.4%
- Oncology = 4 = 8.5%
- Pathology = 0 = 0%
- Reproductive medicine = 2 = 4.3%
- Sports medicine and rehabilitation = 2 = 4.3%
- Animal welfare = 2 = 4.3%
- Behavioral Medicine = 1 = 2.1%
- Birds/exotics = 1 = 2.1%
- Lab animals = 0 = 0%
- Zoo animals = 0 = 0%
- dentistry = 8 = 17.0%
- Other = 11 = 23.4%

**12. Dog owned**

- n = 395
- missing = 0
- yes = 263 = 66.6%
- no = 128 = 32.4%
- I don't want to specify = 4 = 1.0%

**13. Brachycephalic dog owned**

- n = 263
- missing = 0
- yes = 18 = 6.8%
- no = 244 = 92.8%
- I don't want to specify = 1 = 0.4%
