# Supplementary material for: Veterinarians’ attitudes towards the treatment and surgical management of brachycephalic dogs – a survey among small animal veterinarians in Austria
Source: BMC Vet Res. 2026 Jul 1;22:594. doi: 10.1186/s12917-026-05680-9 (PMC13656368; doi:10.1186/s12917-026-05680-9)
Supplement: Supplementary file 2 [file 12917_2026_5680_MOESM2_ESM.docx]

**Colour code:**

Marked in green: Questions aimed exclusively at small animal veterinarians

Purple marked: Instructions for programming the questionnaire

**Questionnaire**

Thank you for participating in this survey.

The aim of the survey is to create an empirical data basis on the attitudes of Austrian veterinarians towards brachycephaly in dogs. The questionnaire is aimed at all veterinarians, but contains separate sections for those participants who work in small animal medicine and treat brachycephalic dogs in their everyday work. Answering the questions will take about 20 minutes.

The study is being carried out as part of a diploma thesis at the Messerli Research Institute of the University of Veterinary Medicine Vienna and has been approved by an ethics committee.

Participation in this survey is voluntary and you can leave it at any time. Your answers will be forwarded to the research team in anonymized form. No conclusions can be drawn about your identity from the information.

By clicking "Continue", you confirm that you are over 17 years of age and agree to participate in this survey.

We thank you in advance for your valuable contribution!

**Section 1: Socio-demographic and practice-specific data collection**

*To begin, we will ask you to provide some demographic information as well as information about your professional career and work environment.*

1. Please indicate your age. (drop-down list from <23 to 100 years incl. "I don't want to specify"; SINGLE CHOICE)

_______Years

2. Please indicate your gender. (SINGLE CHOICE)

a. Male

b. Female

c. Diverse

d. I don't want to specify.

3. What is your current employment status? (MULTIPLE CHOICE)

a. Self-employed, full-time

b. Self-employed, part-time

c. Employed, full-time

d. Employed, part-time

e. I am retired

If e is ticked, a text field appears: "In the following, the questions are formulated for people who are still working. We ask you to answer them with regard to your past professional activity in veterinary medicine."

4. How many years have you been working as a veterinarian? (drop-down list from <0.5 to 80 years; SINGLE CHOICE)

_______Year(s)

5. In which institution(s) do you work? (MULTIPLE CHOICE)

a. Veterinary practice

b. Veterinary clinic

c. University Hospital

d. Mixed practice/clinic

e. Animal shelter as an employee

f. Charity

g. Other

6. In which area (degree of urbanization) is the practice/clinic in which you work registered? If you work in several institutions, please provide information about the institution with the highest number of veterinarians. (SINGLE CHOICE)
a. Large urban center ( > = 100,000 inhabitants)
b. Medium-sized urban center (> = 30,000 and < 100,000 inhabitants)
c. Small urban center (< 30,000 inhabitants)
d. Rural area central (access to urban center < 30 minutes)
e. Rural area peripheral (accessibility to urban center > 30 minutes)
f. I don't know.
7. How many veterinarians work in the practice/clinic where you work (including you)? If you work in several institutions, please provide information about the institution with the highest number of veterinarians. (Dropdown list from 1 to >20 veterinarians; SINGLE CHOICE)

_______ veterinarians

8. In which areas of work do you practice as a veterinarian? Please tick all applicable areas. (MULTIPLE CHOICE)

| Small animal medicine |  |
| --- | --- |
| Livestock medicine |  |
| Equine medicine |  |
| Laboratory Veterinary Medicine |  |
| Zoo Veterinary Medicine |  |
| Exotic medicine |  |
| Avian medicine |  |
| Agency |  |
| Food hygiene and food safety |  |
| Pharmaceutical industry |  |
| Other |  |

9. What percentage of your working time do you work in the field of small animal medicine? (FOLLOW UP QUESTION if "Small Animal Medicine" is ticked at 8.)

| 0% | 1-10% | 11-20% | 21-30% | 31-40% | 41-50% | 51-60% | 61-70% | 71-80% | 81-90% | 91-99% | 100% |
| --- | --- | --- | --- | --- | --- | --- | --- | --- | --- | --- | --- |
|  |  |  |  |  |  |  |  |  |  |  |  |

10. In addition to your degree in veterinary medicine, do you have any of the following qualifications in the field of small animal medicine? Please tick all applicable answers (FOLLOW UP QUESTION, if at least 50% of small animal medicine is ticked at 9., MULTIPLE CHOICE)

a. Specialist veterinarian

b. Diplomate (e.g. European or American College)

c. Doctor of Veterinary Medicine

d. PhD

e. Other

f. I don't have any of the above qualifications.

11. In which department(s) did you specialize? Please tick all applicable answers (FOLLOW UP QUESTION, if at 10. a or b is ticked, MULTIPLE CHOICE)

a. Anaesthesia and pain management

b. Ophthalmology

c. Diagnostic imaging

d. Surgery (orthopaedics)

e. Surgery (soft tissues)

f. Dermatology

g. Internal medicine

h. Cardiology

i. Neurology

j. Oncology

k. Pathology

l. Reproductive medicine

m. Sports Medicine and Rehabilitation

n. Animal welfare

o. Behavioral Medicine

p. Birds/Exotics

q. Laboratory animals

r. Zoo animals

s. Dentistry

t. Other

12. Do you keep one (several) dog(s)? (SINGLE CHOICE)
    a. Yes
    b. No
    c. I don't want to specify.
13. Do you keep one (several) brachycephalic dog(s)? (FOLLOW UP QUESTION, if ticked at 12. a., SINGLE CHOICE)
    a. Yes
    b. No
    c. I don't want to specify.

The entire section 2 including section 2.1 only appears as FOLLOW UP if at least 50% of small animal medicine is ticked at 8.

**Section 2: Medical Care of Brachycephalic Dogs**

The second section of the survey focuses on health problems of brachycephalic dogs and their treatment.

14. What percentage of your patients are dogs in your work as a small animal veterinarian? (FOLLOW UP QUESTION, if at least 50% of small animal medicine is ticked at 9., SINGLE CHOICE)

| 0% | 1-10% | 11-20% | 21-30% | 31-40% | 41-50% | 51-60% | 61-70% | 71-80% | 81-90% | 91-99% | 100% | I do not know |
| --- | --- | --- | --- | --- | --- | --- | --- | --- | --- | --- | --- | --- |
|  |  |  |  |  |  |  |  |  |  |  |  |  |

15. What percentage of the dogs that are presented to you as patients in your work are brachycephalic? (FOLLOW UP QUESTION, if not 0% is ticked at 14., SINGLE CHOICE)

| 0% | 1-10% | 11-20% | 21-30% | 31-40% | 41-50% | 51-60% | 61-70% | 71-80% | 81-90% | 91-99% | 100% | I do not know |
| --- | --- | --- | --- | --- | --- | --- | --- | --- | --- | --- | --- | --- |
|  |  |  |  |  |  |  |  |  |  |  |  |  |

16. In the last five years... (FOLLOW UP QUESTION, if at least 50% of small animal medicine is ticked at 9., SINGLE CHOICE)
a. … the number of brachycephalic dogs presented has increased.
b. … the number of brachycephalic dogs presented has remained the same.
c. … the number of brachycephalic dogs presented has decreased.
d. I don‘t know.
17. To what extent do you agree with the following statements? (FOLLOW UP QUESTION, if at least 50% is ticked for small animal medicine at 9.)

1 = Strongly disagree; 2 = Disagree; 3 = Agree little; 4 = Neutral (neither agreement nor disagreement); 5 = Largely agree; 6 = Agree; 7 = Totally agree; 8 = I don't know;

| **The treatment of a brachycephalic dog...** | 1 | 2 | 3 | 4 | 5 | 6 | 7 | 8 |
| --- | --- | --- | --- | --- | --- | --- | --- | --- |
| … is interesting for me from a medical perspective. |  |  |  |  |  |  |  |  |
| … causes a lack of understanding about owning such a dog. |  |  |  |  |  |  |  |  |
| … triggers anger in me about owning such a dog. |  |  |  |  |  |  |  |  |
| … is not different for me from the treatment of other dogs. |  |  |  |  |  |  |  |  |

18. How often are the following health problems a reason for treatment in brachycephalic dogs? (FOLLOW UP QUESTION if 0% is not ticked at 15.)

|  | Not at all | Seldom | Occasio-nally | Frequen-tly | Very common | I do not know |
| --- | --- | --- | --- | --- | --- | --- |
| Shortness of breath |  |  |  |  |  |  |
| Movement abnormalities |  |  |  |  |  |  |
| Lameness |  |  |  |  |  |  |
| Inflammation of the skin |  |  |  |  |  |  |
| Baldness |  |  |  |  |  |  |
| Inflammation of the conjunctiva and/or cornea |  |  |  |  |  |  |
| Blindness |  |  |  |  |  |  |
| Exophthalmos |  |  |  |  |  |  |
| Deafness |  |  |  |  |  |  |
| Neurological symptoms |  |  |  |  |  |  |
| Malformations of the teeth |  |  |  |  |  |  |
| Malformations of the skull |  |  |  |  |  |  |
| Gastrointestinal symptoms |  |  |  |  |  |  |
| Body shapes in which it must be assumed with a high degree of probability that natural births are not possible |  |  |  |  |  |  |

**Section 2.1: Brachycephalic Obstructive Airway Syndrome (BOAS)**

*Brachycephalic obstructive airway syndrome, or BOAS for short, refers to functional changes that occur in some brachycephalic dogs due to the shortened facial skull with non-proportionally reduced soft tissue. Typical symptoms of BOAS are, for example, difficulty breathing, reduced exercise capacity, heat intolerance, hyperthermia, cyanosis, regular collapse, sleep disorders, snoring or gagging, as well as gastrointestinal symptoms. There are currently two established methods for BOAS screening: on the one hand, the performance of an exercise tolerance test, and on the other hand, the assessment of morphological features, such as the calculation of the craniofacial ratio by dividing the skull length by the snout length.*

*In the following, we ask you for information about your observation of BOAS symptoms in brachycephalic dogs. In addition, the possibility of corrective surgery to alleviate BOAS symptoms is discussed in more detail.*

19. In what percentage of the brachycephalic dogs that you care for as patients are symptoms of BOAS the main reason for presentation? (FOLLOW UP QUESTION, if not 0% is ticked at 15., SINGLE CHOICE)

| 0% | 1-10% | 11-20% | 21-30% | 31-40% | 41-50% | 51-60% | 61-70% | 71-80% | 81-90% | 91-99% | 100% | I do not know |
| --- | --- | --- | --- | --- | --- | --- | --- | --- | --- | --- | --- | --- |
|  |  |  |  |  |  |  |  |  |  |  |  |  |

20. How often do the following symptoms of BOAS occur in the brachycephalic dogs you care for as patients? (FOLLOW UP QUESTION if 0% is not ticked at 15.)

|  | Not at all | Seldom | Occasio-nally | Frequen-tly | Very common | I do not know. |
| --- | --- | --- | --- | --- | --- | --- |
| Difficulty breathing |  |  |  |  |  |  |
| Reduced exercise capacity |  |  |  |  |  |  |
| Heat intolerance |  |  |  |  |  |  |
| Hyperthermia |  |  |  |  |  |  |
| Cyanosis |  |  |  |  |  |  |
| Regular collapse |  |  |  |  |  |  |
| Insomnia |  |  |  |  |  |  |
| Snoring |  |  |  |  |  |  |
| Gagging and swallowing disorders |  |  |  |  |  |  |
| Gastrointestinal symptoms |  |  |  |  |  |  |

21. What percentage of brachycephalic dogs with symptoms of BOAS that you care for as patients do you recommend surgery to relieve symptoms? (FOLLOW UP QUESTION, if not 0% is ticked at 15., SINGLE CHOICE)

| 0% | 1-10% | 11-20% | 21-30% | 31-40% | 41-50% | 51-60% | 61-70% | 71-80% | 81-90% | 91-99% | 100% | I do not know |
| --- | --- | --- | --- | --- | --- | --- | --- | --- | --- | --- | --- | --- |
|  |  |  |  |  |  |  |  |  |  |  |  |  |

22. What percentage of brachycephalic dogs with symptoms of BOAS for whom you recommend surgery actually undergo surgery? (FOLLOW UP QUESTION, if not 0% is ticked at 21., SINGLE CHOICE)

| 0% | 1-10% | 11-20% | 21-30% | 31-40% | 41-50% | 51-60% | 61-70% | 71-80% | 81-90% | 91-99% | 100% | I do not know |
| --- | --- | --- | --- | --- | --- | --- | --- | --- | --- | --- | --- | --- |
|  |  |  |  |  |  |  |  |  |  |  |  |  |

23. Where are surgeries performed to relieve symptoms of BOAS of your patients? (FOLLOW UP QUESTION, if at 22. 0% is not ticked, MULTIPLE CHOICE)
    a. I perform the surgeries myself in the practice/clinic where I work.
    b. One of my colleagues performs the surgery in the practice/clinic where I work.
    c. I refer the patient to another practice/clinic where the surgery is performed.
    d. Other
24. If pet owners reject a surgery you recommend to alleviate BOAS symptoms, to what extent do the following factors influence the rejection on the part of the pet owners? (FOLLOW UP QUESTION if 100% is not ticked at 22.)

|  | Not at all | Very little | Moderate | Rather strong | Very strong | I don't know. |
| --- | --- | --- | --- | --- | --- | --- |
| Limited financial resources |  |  |  |  |  |  |
| Weak emotional attachment |  |  |  |  |  |  |
| Limited time resources to care for the animal |  |  |  |  |  |  |
| Fear of complications |  |  |  |  |  |  |
| Doubts about the success of the operation |  |  |  |  |  |  |
| Doubts about the necessity of the operation |  |  |  |  |  |  |
| Negative experiences with known animals that have received this therapy |  |  |  |  |  |  |
| Negative reports about the treatment in the press and on social media |  |  |  |  |  |  |

**Case vignettes**

*For the case vignettes an experimental approach is chosen. The study population will be randomized into thirds and confronted with different cases.*

**Group A – BRANCH A 33%**

25. A dog owner comes to you with her two-year-old pug Maxi. Maxi's difficulty breathing is immediately noticeable. In addition, the dog owner reports that she can hardly go out of the house with Maxi on warm days and that he **snores terribly loudly while** sleeping. Based on the symptoms and further diagnostics, you will diagnose BOAS in Maxi. How would you proceed? (FOLLOW UP QUESTION, if at least 50% of small animal medicine is ticked for 9., SINGLE CHOICE)
    a. I would recommend surgery to relieve the symptoms of BOAS.
    b. I would not recommend any surgical intervention to relieve the symptoms of BOAS.
    c. Other
26. Please move each of the following reasons to the appropriate category that reflects best how important that reason was for your decision. (FOLLOW UP QUESTION, if a. is ticked at 25.)

| Reason 1 | I have had positive experiences with the surgical correction of BOAS. |
| --- | --- |
| Reason 2 | I have heard of positive results from the surgical correction of BOAS. |
| Reason 3 | I think that any available treatment should be tried. |
| Reason 4 | The patient is still very young. |
| Reason 5 | The symptom of snoring is a reason to recommend surgery. |
| Reason 6 | The symptom of load incapacity is a reason to recommend surgery |

| Not important at all | Less important | Important | Very important | I do not know. |
| --- | --- | --- | --- | --- |
|  |  |  |  |  |

27. Please move each of the following reasons to the appropriate category that reflects best how important that reason was for your decision. (FOLLOW UP QUESTION if b. is ticked at 25.)

| Reason 1 | I have had negative experiences with the surgical correction of BOAS. |
| --- | --- |
| Reason 2 | I have heard of negative results from the surgical correction of BOAS. |
| Reason 3 | Surgical correction of BOAS involves many risks and side effects. |
| Reason 4 | A surgical correction of BOAS causes high costs for the animal owner. |
| Reason 5 | The symptom of snoring is no reason to recommend surgery. |
| Reason 6 | The symptom of load incapacity is no reason to recommend surgery. |

| Not important at all | Less important | Important | Very important | I do not know. |
| --- | --- | --- | --- | --- |
|  |  |  |  |  |

**Group B – BRANCH B 33%**

28. A dog owner comes to you with her two-year-old pug Maxi. Maxi's difficulty breathing is immediately noticeable. In addition, the dog owner reports that she can hardly go out of the house with Maxi on warm days and that **Maxi's tongue even turns blue during exertion.** Based on the symptoms and further diagnostics, you will diagnose BOAS in Maxi. How would you proceed? (FOLLOW UP QUESTION, if at least 50% of small animal medicine is ticked for 9, SINGLE CHOICE)
    a. I would recommend surgery to relieve the symptoms of BOAS.
    b. I would not recommend any surgical intervention to relieve the symptoms of BOAS.
    c. Other
29. Please move each of the following reasons to the appropriate category that reflects best how important that reason was for your decision. (FOLLOW UP QUESTION if a. is ticked at 28.)

| Reason 1 | I have had positive experiences with the surgical correction of BOAS. |
| --- | --- |
| Reason 2 | I have heard of positive results from the surgical correction of BOAS. |
| Reason 3 | I think that any available treatment should be tried. |
| Reason 4 | The patient is still very young. |
| Reason 5 | The patient's cyanosis is a reason to recommend surgery. |
| Reason 6 | The symptom of load incapacity is a reason to recommend surgery. |

| Not important at all | Less important | Important | Very important | I do not know. |
| --- | --- | --- | --- | --- |
|  |  |  |  |  |

30. Please move each of the following reasons to the appropriate category that reflects best how important that reason was for your decision. (FOLLOW UP QUESTION if b. is ticked at 28.)

| Reason 1 | I have had negative experiences with the surgical correction of BOAS. |
| --- | --- |
| Reason 2 | I have heard of negative results from the surgical correction of BOAS. |
| Reason 3 | Surgical correction of BOAS involves many risks and side effects. |
| Reason 4 | A surgical correction of BOAS causes high costs for the animal owner. |
| Reason 5 | The patient's cyanosis is not a reason to recommend surgery. |
| Reason 6 | The symptom of load incapacity is no reason to recommend surgery. |

| Not important at all | Less important | Important | Very important | I do not know. |
| --- | --- | --- | --- | --- |
|  |  |  |  |  |

**Group C – BRANCH C 34%**

31. A dog owner comes to you with her two-year-old pug Maxi. Maxi's difficulty breathing is immediately noticeable. In addition, the dog owner reports that she can hardly go out of the house with Maxi on warm days. **While walking, he had already collapsed several times exhausted and even lost consciousness the last time.** Based on the symptoms and further diagnostics, you will diagnose BOAS in Maxi. How would you proceed? (FOLLOW UP QUESTION, if at least 50% of small animal medicine is ticked for 9., SINGLE CHOICE)
    a. I would recommend surgery to relieve the symptoms of BOAS.
    b. I would not recommend any surgical intervention to relieve the symptoms of BOAS.
    c. Other
32. Please move each of the following reasons to the appropriate category that reflects best how important that reason was for your decision. (FOLLOW UP QUESTION if a. is ticked at 31.)

| Reason 1 | I have had positive experiences with the surgical correction of BOAS. |
| --- | --- |
| Reason 2 | I have heard of positive results from the surgical correction of BOAS. |
| Reason 3 | I think that any available treatment should be tried. |
| Reason 4 | The patient is still very young. |
| Reason 5 | The repeated collapse of the patient is a reason to recommend surgery. |
| Reason 6 | The patient's loss of consciousness is a reason to recommend surgery. |
| Reason 7 | The symptom of load incapacity is a reason to recommend surgery. |

| Not important at all | Less important | Important | Very important | I do not know. |
| --- | --- | --- | --- | --- |
|  |  |  |  |  |

33. Please move each of the following reasons to the appropriate category that reflects best how important that reason was for your decision. (FOLLOW UP QUESTION if b. is ticked 31.)

| Reason 1 | I have had negative experiences with the surgical correction of BOAS. |
| --- | --- |
| Reason 2 | I have heard of negative results from the surgical correction of BOAS. |
| Reason 3 | Surgical correction of BOAS involves many risks and side effects. |
| Reason 4 | A surgical correction of BOAS causes high costs for the animal owner. |
| Reason 5 | The repeated collapse of the patient is not a reason to recommend surgery. |
| Reason 6 | The patient's loss of consciousness is not a reason to recommend surgery. |
| Reason 7 | The symptom of load incapacity is no reason to recommend surgery. |

| Not important at all | Less important | Important | Very important | I do not know. |
| --- | --- | --- | --- | --- |
|  |  |  |  |  |

**Section 3: Assessments of buying a brachycephalic dog**

*In this section, we ask you for your assessment of pet owners' reasons for choosing a brachycephalic dog and their level of information on possible breed-specific problems.*

34. To what extent do the following factors influence pet owners to choose a brachycephalic dog?

|  | Not at all | Very little | Moderate | Rather strong | Very strong | I do not know. |
| --- | --- | --- | --- | --- | --- | --- |
| Character |  |  |  |  |  |  |
| Size |  |  |  |  |  |  |
| Characteristic appearance of the breeds |  |  |  |  |  |  |
| Activity Level |  |  |  |  |  |  |
| Need for care |  |  |  |  |  |  |
| Status symbol |  |  |  |  |  |  |
| Family-friendliness |  |  |  |  |  |  |
| Snoring/grunting |  |  |  |  |  |  |
| Lack of information |  |  |  |  |  |  |

35. How often are you as a veterinarian visited by a pet owner for an informational talk before buying a brachycephalic dog? (FOLLOW UP QUESTION, if at least 50% of small animal medicine is ticked in 9., SINGLE CHOICE)
a. never
b. very rare
c. seldom
d. every now and then
e. frequently
f. very common
36. A potential dog owner comes to you for an informational meeting before buying a puppy. He reports that he would like to buy a pug. Please drag each of the following into the appropriate category that reflects best how likely it would be to match your response. (FOLLOW UP QUESTION, if at least 50% is ticked for small animal medicine at 9.)

| Reaction 1 | I would advise him against his election. |
| --- | --- |
| Reaction 2 | I would suggest other dog breeds that are healthier on average. |
| Reaction 3 | I would tell him that he has chosen an extremely lovable breed. |
| Reaction 4 | I would not comment on the choice of breed. |
| Reaction 5 | I would explain to him in detail what he can look out for when buying a puppy in order to acquire the healthiest pug possible. |

| Very unlikely | Rather unlikely | Rather likely | Very likely |
| --- | --- | --- | --- |
|  |  |  |  |

37. What percentage of the brachycephalic dogs that you care for as patients have health insurance? (FOLLOW UP QUESTION if 0% is not ticked at 15.)

| 0% | 1-10% | 11-20% | 21-30% | 31-40% | 41-50% | 51-60% | 61-70% | 71-80% | 81-90% | 91-99% | 100% | I do not know |
| --- | --- | --- | --- | --- | --- | --- | --- | --- | --- | --- | --- | --- |
|  |  |  |  |  |  |  |  |  |  |  |  |  |

38. To what extent do you agree with the following statements? (FOLLOW UP QUESTION, if at least 50% is ticked for small animal medicine at 9.)

1 = Strongly disagree; 2 = Disagree; 3 = Agree little; 4 = Neutral (neither agreement nor disagreement); 5 = Largely agree; 6 = Agree; 7 = Totally agree; 8 = I don't know;

| **Owners of brachycephalic dogs...** | 1 | 2 | 3 | 4 | 5 | 6 | 7 | 8 |
| --- | --- | --- | --- | --- | --- | --- | --- | --- |
| … usually have a closer bond with their animals than owners of other dog breeds. |  |  |  |  |  |  |  |  |
| … are often overwhelmed when their animals get sick. |  |  |  |  |  |  |  |  |
| … often underestimate the possible symptoms of brachycephaly. |  |  |  |  |  |  |  |  |
| … are usually not sufficiently informed about possible breed-specific symptoms. |  |  |  |  |  |  |  |  |
| … often do not recognize breed-specific symptoms. |  |  |  |  |  |  |  |  |
| … see symptoms of brachycephaly as "normal" for the breed. |  |  |  |  |  |  |  |  |
| … underestimate the veterinary costs that can arise as a result of brachycephaly. |  |  |  |  |  |  |  |  |
| ... are financially able to fully bear veterinary costs caused by breed-specific symptoms. |  |  |  |  |  |  |  |  |
| … I would advise taking a pet health insurance. |  |  |  |  |  |  |  |  |

**Section 4: Breeding and handling of brachycephalic dogs**

*This section of the questionnaire deals with the breeding and handling of brachycephalic dogs, as well as the topic of short-headed dogs on an Austrian and EU-wide level.*

39. To what extent do you agree with the following statements?

1 = Strongly disagree; 2 = Disagree; 3 = Agree little; 4 = Neutral (neither agreement nor disagreement); 5 = Largely agree; 6 = Agree; 7 = Totally agree; 8 = I don't know;

| **A mandatory step before the breeding approval of brachycephalic dogs should be...** | 1 | 2 | 3 | 4 | 5 | 6 | 7 | 8 |
| --- | --- | --- | --- | --- | --- | --- | --- | --- |
| … the performance of a BOAS load test. |  |  |  |  |  |  |  |  |
| … the performance of an objective assessment of certain morphological features, such as the size of the nostrils, by a trained professional. |  |  |  |  |  |  |  |  |
| … the calculation of the craniofacial ratio by dimensions of the skull. |  |  |  |  |  |  |  |  |

40. To what extent do you agree with the following statements?

1 = Strongly disagree; 2 = Disagree; 3 = Agree little; 4 = Neutral (neither agreement nor disagreement); 5 = Largely agree; 6 = Agree; 7 = Totally agree; 8 = I don't know;

| **The exclusion of brachycephalic dogs from breeding on the basis of defined criteria...** | 1 | 2 | 3 | 4 | 5 | 6 | 7 | 8 |
| --- | --- | --- | --- | --- | --- | --- | --- | --- |
| … would reduce the gene pool for breeding too much. |  |  |  |  |  |  |  |  |
| … is the only way to breed healthier offspring. |  |  |  |  |  |  |  |  |
| … makes sense if long-snouted dog breeds are crossed instead of the excluded subpopulation. |  |  |  |  |  |  |  |  |

41. To what extent do you agree with the following statements?

1 = Strongly disagree; 2 = Disagree; 3 = Agree little; 4 = Neutral (neither agreement nor disagreement); 5 = Largely agree; 6 = Agree; 7 = Totally agree; 8 = I don't know;

| **Regarding breeding with brachycephalic dogs...** | 1 | 2 | 3 | 4 | 5 | 6 | 7 | 8 |
| --- | --- | --- | --- | --- | --- | --- | --- | --- |
| … a fixed date should be determined in Austria by which a defined breeding goal must be achieved. |  |  |  |  |  |  |  |  |
| … an EU-wide regulation on the subject of breeding makes more sense than the current regulations at national level. |  |  |  |  |  |  |  |  |
| … a mandatory registration of all dogs kept as pets in the EU is urgently needed in order to be able to monitor dog breeding. |  |  |  |  |  |  |  |  |
| … it is necessary to ban the ownership of certain dog breeds throughout the EU. |  |  |  |  |  |  |  |  |
| … it is necessary to ban certain breeding characteristics throughout the EU. |  |  |  |  |  |  |  |  |

**Section 5: Education about brachycephaly in dogs**

*Finally, there is an opinion poll on the topic of education regarding brachycephaly in dogs.*

42. To what extent do you agree with the following statements?

1 = Strongly disagree; 2 = Disagree; 3 = Agree little; 4 = Neutral (neither agreement nor disagreement); 5 = Largely agree; 6 = Agree; 7 = Totally agree; 8 = I don't know;

| **It would be good that...** | 1 | 2 | 3 | 4 | 5 | 6 | 7 | 8 |
| --- | --- | --- | --- | --- | --- | --- | --- | --- |
| … the problem is already addressed in schools. |  |  |  |  |  |  |  |  |
| … there are more public campaigns on the subject of brachycephaly. |  |  |  |  |  |  |  |  |
| … there are more public discussions on the subject of brachycephaly. |  |  |  |  |  |  |  |  |
| … more veterinarians address the issue of brachycephaly to pet owners of brachycephalic dogs. |  |  |  |  |  |  |  |  |
| … more veterinarians contribute to the public discussion on the topic of brachycephaly. |  |  |  |  |  |  |  |  |
| … more veterinarians offer consultations before acquiring a brachycephalic dog. |  |  |  |  |  |  |  |  |
| … veterinarians and breeders of brachycephalic dogs work more closely together. |  |  |  |  |  |  |  |  |
