# Supplementary material for: Veterinarians’ attitudes towards the treatment and surgical management of brachycephalic dogs – a survey among small animal veterinarians in Austria
Source: BMC Vet Res. 2026 Jul 1;22:594. doi: 10.1186/s12917-026-05680-9 (PMC13656368; doi:10.1186/s12917-026-05680-9)
Supplement: Supplementary file 3 [file 12917_2026_5680_MOESM3_ESM.docx]

**Ordinal logistic regression analysis to examine the effect of socio-demographic and practice-related aspects on the veterinarians’ perception of treating brachycephalic dogs**

| **1** | **… is interesting for me from a medical perspective.**  (χ^2^(6)=10.989, P=0.089) | | | | | |
| --- | --- | --- | --- | --- | --- | --- |
| **2** | **… causes a lack of understanding about owning such a dog**  (χ^2^(6)=16.596, P=0.011) | | | | | |
|  |  | **B** | **Std. Error** | **Wald Chi-Square** | **df** | **Sig.** |
|  | gender | -0.027 | 0.4144 | 0.004 | 1 | 0.949 |
|  | Level of urbanization | -0.148 | 0.2832 | 0.275 | 1 | 0.600 |
|  | Performance of procedure | 0.284 | 0.2943 | 0.928 | 1 | 0.335 |
|  | Owning a dog | -0.540 | 0.2997 | 3.252 | 1 | 0.071 |
|  | Work experience | -0.041 | 0.0133 | 9.495 | 1 | 0.002 |
|  | Percentage of brachycephalic patients | -0.143 | 0.1172 | 1.485 | 1 | 0.223 |
| **3** | **… triggers anger in me about owning such a dog.**  (χ^2^(6)=11.816, P=0.066) | | | | | |
| **4** | **… triggers anger in me about owning such a dog.**  (χ^2^(6)=0.949, P=0.987) | | | | | |
