# Supplementary material for: Veterinarians’ attitudes towards the treatment and surgical management of brachycephalic dogs – a survey among small animal veterinarians in Austria
Source: BMC Vet Res. 2026 Jul 1;22:594. doi: 10.1186/s12917-026-05680-9 (PMC13656368; doi:10.1186/s12917-026-05680-9)
Supplement: Supplementary file 4 [file 12917_2026_5680_MOESM4_ESM.docx]

Ordinal logistic regression analysis to examine the effect of veterinarian-related factors on **the frequency of recommending surgical intervention** to alleviate BOAS signs

| (χ^2^(8)=24.021, P=0.002) | | | | | |
| --- | --- | --- | --- | --- | --- |
|  | **B** | **Std. Error** | **Wald Chi-Square** | **df** | **Sig.** |
| Work experience | -,023 | ,012 | 3,701 | 1 | ,054 |
| gender | -,125 | ,361 | ,119 | 1 | ,730 |
| Level of urbanization | ,356 | ,264 | 1,824 | 1 | ,177 |
| Performance of surgical intervention | ,966 | ,271 | 12,745 | 1 | <,001 |
| level of agreement towards the statement “The treatment of a brachycephalic dog is interesting from a medical point of view” (Disgreement) *(ref. neutral/I don’t know)* | -,065 | ,280 | ,054 | 1 | ,817 |
| level of agreement towards the statement “The treatment of a brachycephalic dog is interesting from a medical point of view” (Agreement *(ref. neutral/I don’t know)* | ,112 | ,351 | ,102 | 1 | ,749 |
| level of agreement towards the statement “The treatment of a brachycephalic dog does not differ from the treatment of other dogs” (Disagreement) *(ref. neutral/I don’t know)* | -,306 | ,326 | ,878 | 1 | ,349 |
| level of agreement towards the statement “The treatment of a brachycephalic dog does not differ from the treatment of other dogs” (Agreement *(ref. neutral/I don’t know)* | -,508 | ,355 | 2,052 | 1 | ,152 |

Ordinal logistic regression analysis to examine the effect of veterinarian-related factors on **the frequency of actual surgical intervention** based on the veterinarians’ recommendation to alleviate BOAS signs

| (χ^2^(8)=24.021, P=0.002) | | | | | |
| --- | --- | --- | --- | --- | --- |
|  | **B** | **Std. Error** | **Wald Chi-Square** | **df** | **Sig.** |
| Work experience | ,045 | ,015 | 9,043 | 1 | ,003 |
| gender | 1,175 | ,412 | 8,113 | 1 | ,004 |
| Level of urbanization | ,169 | ,321 | ,276 | 1 | ,599 |
| Performance of surgical intervention | 1,304 | ,315 | 17,181 | 1 | <,001 |
| level of agreement towards the statement “The treatment of a brachycephalic dog is interesting from a medical point of view” (Disgreement) *(ref. neutral/I don’t know)* | -,060 | ,336 | ,032 | 1 | ,857 |
| level of agreement towards the statement “The treatment of a brachycephalic dog is interesting from a medical point of view” (Agreement *(ref. neutral/I don’t know)* | ,231 | ,401 | ,333 | 1 | ,564 |
| level of agreement towards the statement “The treatment of a brachycephalic dog does not differ from the treatment of other dogs” (Disagreement) *(ref. neutral/I don’t know)* | -,036 | ,381 | ,009 | 1 | ,924 |
| level of agreement towards the statement “The treatment of a brachycephalic dog does not differ from the treatment of other dogs” (Agreement *(ref. neutral/I don’t know)* | -,005 | ,423 | ,000 | 1 | ,990 |
